# Lung in Dengue: Computed Tomography Findings

**DOI:** 10.1371/journal.pone.0096313

**Published:** 2014-05-16

**Authors:** Rosana Souza Rodrigues, Ana Livia Garcia Brum, Marciano Viana Paes, Tiago Fajardo Póvoa, Carlos Alberto Basilio-de-Oliveira, Edson Marchiori, Danielle Provençano Borghi, Grazielle Viana Ramos, Fernando Augusto Bozza

**Affiliations:** 1 D’Or Institute for Research and Education, Rio de Janeiro/RJ, Brazil; 2 Federal University of Rio de Janeiro, Rio de Janeiro/RJ, Brazil; 3 Biotecnology and Phisiology of Viral Infection, Instituto Oswaldo Cruz, Fundação Oswaldo Cruz, Rio de Janeiro/RJ, Brazil; 4 Federal University of State of Rio de Janeiro, Rio de Janeiro/RJ, Brazil; 5 Corpo de Bombeiros Militar do Estado do Rio de Janeiro, Rio de Janeiro/RJ, Brazil; 6 Instituto de Pesquisa Clínica Evandro Chagas (IPEC), Fundação Oswaldo Cruz, Rio de Janeiro/RJ, Brazil; University Hospital San Giovanni Battista di Torino, Italy

## Abstract

**Background:**

Dengue is the most important mosquito-borne viral disease in the world. Dengue virus infection may be asymptomatic or lead to undifferentiated fever, dengue fever with or without warning signs, or severe dengue. Lower respiratory symptoms are unusual and lung-imaging data in patients with dengue are scarce.

**Methodology/Principal Findings:**

To evaluate lung changes associated with dengue infection, we retrospectively analyzed 2,020 confirmed cases of dengue. Twenty-nine of these patients (11 females and 18 males aged 16–90 years) underwent chest computed tomography (CT), which yielded abnormal findings in 17 patients: 16 patients had pleural effusion (the sole finding in six patients) and 11 patients had pulmonary abnormalities. Lung parenchyma involvement ranged from subtle to moderate unilateral and bilateral abnormalities. The most common finding was ground-glass opacity in eight patients, followed by consolidation in six patients. Less common findings were airspace nodules (two patients), interlobular septal thickening (two patients), and peribronchovascular interstitial thickening (one patient). Lung histopathological findings in four fatal cases showed thickening of the alveolar septa, hemorrhage, and interstitial edema.

**Conclusions/Significance:**

In this largest series involving the use of chest CT to evaluate lung involvement in patients with dengue, CT findings of lower respiratory tract involvement were uncommon. When abnormalities were present, pleural effusion was the most frequent finding and lung involvement was often mild or moderate and bilateral. Extensive lung abnormalities are infrequent even in severe disease and when present should lead physicians to consider other diagnostic possibilities.

## Introduction

Dengue is the most important arthropod-borne viral infection in humans. Rapid expansion of the numbers of cases and countries affected by the disease has been observed in recent years, rendering dengue the most significant re-emerging infectious disease, especially in tropical and subtropical regions [1]. Recently, it was estimated there to be 390 million dengue infections per year, of which 96 million manifest apparently (any level of clinical or sub-clinical severity) [2]. The global resurgence of dengue is thought to be due to many factors, including failure to control *Aedes* populations, uncontrolled urbanization, population growth, climate change, and an increasing number of international travelers [3].

In the Americas, the disease resurged after the 1980s, with cyclical outbreaks occurring every 3–5 years [4]. In past years, dengue outbreaks in Brazil entailed at least 1 million cases. Dengue virus (DEN)-3 was formerly the predominant circulating serotype, but the renewed circulation of DEN-2 and DEN-4 has led to an increase in severe dengue (SD) cases, which has imposed significant challenges to the health system, including resource allocation and the early identification and clinical management of severe cases [5].

A new classification of severity levels was recently proposed by a panel of experts and adopted in the World Health Organization (WHO) dengue guidelines [6]. This classification suggests a distinction between dengue and SD, with the presence of severe organ dysfunction serving as one SD criterion. Studies of dengue have produced controversial results with respect to lung involvement. Dengue viral antigens have been demonstrated in lung tissue in experimental infection and autopsy specimens, and the virus appears to potentially infect macrophages and lung endothelial and epithelial cells [7], [8]. However, analyses of the clinical spectrum of lung affection are scare and limited to case reports, and little overall information on chest imaging findings is available [9], [10]. In this study, we evaluated the frequency and pattern of thoracic involvement in serologically confirmed dengue using chest computed tomography (CT), compared the imaging features of dengue fever (DF) and SD, and evaluated autopsy specimens from four patients who died with the disease. To our knowledge, this study is the first systematic examination of chest CT features in this population.

## Materials and Methods

### Ethics Statement

Our institutional clinical research ethics board (Comitê de Ética em Pesquisa Hospital Copa D’Or) approved this retrospective study, for which the informed consent requirement was waived. Ethics committee (Comitê de Ética em Pesquisa – Fundação Oswaldo Cruz -Fiocruz) from the institution that performed histopathological analysis approved the use of autopsy specimens and waived written informed consent from the next of kin. All data used in this study was anonymized.

### Patients

We included all patients aged≥16 years who had clinically suspected dengue and were seen on an inpatient or outpatient basis at three participating hospitals in Rio de Janeiro, Brazil, between January 2007 and December 2008. Diagnoses of dengue fulfilled the WHO case definitions [6] and were confirmed by immunoglobulin M (IgM) positivity. Patients who did not receive reference test diagnoses or refused blood sampling, and those for whom inappropriate or insufficient material was available or serological findings were indeterminate, were excluded. Clinical data were collected retrospectively using a standardized case report form that included demographic and epidemiologic data, clinical presentation, laboratory data, time course of acute illness, need for intensive care, and in-hospital mortality.

Cases were classified according to the 2009 WHO guidelines [6] as DF, dengue with warning signs, or SD. Warning signs were abdominal pain/tenderness, persistent vomiting, fluid accumulation (pleural effusion [PE] or ascites), mucosal bleeding, liver enlargement, and increased hematocrit concurrent with a rapid decrease in the platelet count. The criteria for SD were: severe plasma leakage; severe bleeding; severe organ involvement, comprising hepatic injury (aspartate [AST] or alanine transaminase [ALT] level ≥1000 units/L) and/or renal impairment (serum creatinine increase of 100% over baseline or calculated norm for age/sex/race); and/or impaired consciousness. Patients with no warning sign or criterion for SD were classified as having DF [6].

Blood indices were tabulated and then categorized on the basis of clinically meaningful cutoff values. Thrombocytopenia was defined as a platelet count <150,000 cells/mm^3^ blood. A hematocrit value >48% was considered to be elevated. Leucopenia was defined as a white blood cell count <4000 cells/mm^3^. ALT levels >55 and >33 IU/L and AST levels >46 and >32 IU/L for males and females, respectively, were defined as elevated. Urea values >21 mg/dL and creatinine levels >1.3 and >1.1 mg/dL for males and females, respectively, were considered to be elevated.

### CT Imaging

Chest CT was performed using a multi-detector row scanner (16–64 detectors). The acquisition parameters for CT were 0.625 or 1.25 mm collimation with a pitch of 0.9–1.75, 120 kV, 115–260 mA/s or automatic mA adjustment, and 0.6–0.8 s per gantry rotation. Studies were performed using standard and high-resolution algorithms. Initial chest CT examinations selected to assign CT lesion scores were performed 2–21 (median, 5; mean, 5.8) days after the onset of clinical symptoms. Each patient underwent at least one chest CT examination.

### CT Analysis

Two thoracic radiologists with 4 and 15 years of experience in pulmonary imaging, respectively, reviewed the CT images and reached consensus on their interpretation. All images were reviewed at a window width and level of 1400 and –650 HU, respectively, for lung parenchyma, and 300 and 35 HU, respectively, for mediastinum. The readers recorded the presence of consolidation, ground-glass opacity (GGO), airspace nodules, interlobular septal thickening, and peribronchovascular interstitial thickening. These qualitative descriptions of CT findings were based on the recommendations of the Nomenclature Committee of the Fleischner Society [11]. Atelectasis associated with PE was not recorded.

The extent of consolidation, GGO, and nodules was graded as mild (<25%), moderate (25–75%), or severe (>75%), depending the percentage of the lobe affected. Findings were categorized according to their distributions as diffuse, central, peripheral, or having no specific distribution, as well as by their predominance in the upper, middle, or lower third of the lungs [12]. PE was semiquantitatively scored according to the maximum anteroposterior depth of pleural fluid (measured at the maximum effusion depth, which varied by patient) as small (<15% hemithorax diameter), moderate (15–30%), or large (>30%) [13].

### Histopathological Analysis

In an attempt to better understand the pathological phenomena occurring in the lungs of patients with dengue, autopsy specimens of four patients who died with the disease but did not undergo chest CT were evaluated. Formalin-fixed and paraffin-embedded lung tissues were cut into 4-µm sections, deparaffinized in xylene, and rehydrated with alcohol. These sections were stained with hematoxylin and eosin for histological examination.

### Statistical Analysis

Statistical analyses were performed using GraphPad Prism, version 5.0 for Windows (GraphPad Software, San Diego, CA, USA). Continuous variables were summarized as median and interquartile ranges. We compared the distribution of continuous variables by using the t test, and categoric variables by using the χ^2^ test.

## Results

**Figure 1:**
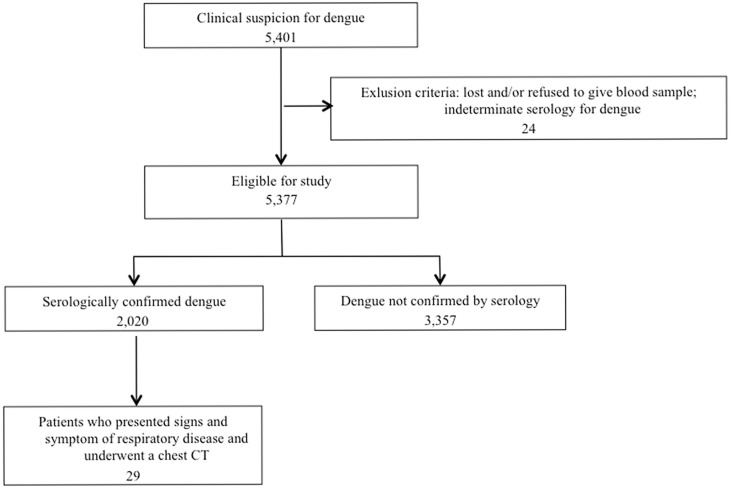
Flowchart of patient selection.

During a 24-month period, 5401 patients with clinical suspicion for dengue were identified; 5377 of these patients were eligible for the study and 2020 had serologically confirmed dengue. Twenty-nine of these patients who presented signs and symptoms of respiratory disease and underwent chest CT formed our study population. Enrollment and exclusion criteria are shown in Fig. 1.

### Clinical Characteristics

The study population comprised 11 (37.93%) females and 18 (62.07%) males aged 16–90 years (mean, 56.8 years; median, 58 years; interquartile range, 35.5%–75%). DF was diagnosed in 9/29 patients (6 males, 3 females) and warning signs or SD (W/SD) were diagnosed in 20/29 patients (12 males, 8 females). Ten patients (22.22% of those with DF and 40% of those with W/SD) had previously had dengue. Seventeen of the 29 (58.62%) patients were treated in the intensive care unit. The length of hospitalization ranged from 1 to 62 (median, 6; mean, 10) days. Unfortunately, 2/29 (6.89%) patients, who had W/SD, died during treatment. Patients’ demographic, clinical, and laboratory features are described in Table 1.

**Table 1:** Demographic, clinical, and laboratorial characteristics of patients with dengue fever (DF), warning signs, and severe dengue (SD).

Characteristic	Dengue Total N = 29	DF N = 9	W/SD N = 20
Age	16–90 (median, 58)	32–86 (median, 58)	16–90 (median, 57)
Gender (M/F)	18/11	6/3	12/8
Previous Dengue	10	2	8
Clinical Presentation			
Fever	25	7	18
Temperature (°C)	39	median, 39	median, 39
Nausea and/or vomiting	10 (34.5%)	0 (0%)	10 (50%)
Abdominal Pain	9 (31.0%)	0 (0%)	9 (45%)
Rash	5 (17.2%)	0 (0%)	5 (25%)
Diarrhea	6 (20.7%)	0 (0%)	6 (30%)
Myalgia	19 (65.5%)	4 (44.4%)	15 (75%)
Headache	14 (48.3%)	1 (11.1%)	13 (65%)
Cough	8 (27.6%)	2 (22.2%)	6 (30%)
Dyspnea	11 (37.9%)	2 (22.2%)	9 (45%)
Drowsiness	15 (51.7%)	2 (22.2%)	13 (65%)
Hemorrhage	12 (41.4%)	0 (0%)	12 (60%)
Received platelet transfusion	2 (6.9%)	0 (0%)	2 (10%)
Laboratory Findings			
Platelets (Platelets/mm^3^)	median, 78000	median, 110000	median, 69000
Hematocrit level on admission (%)	median, 40.6	median, 39	median, 43
Leukocytes (leukocytes/mm^3^)	median, 3400	median, 3600	median, 3200
AST (1000 unit/L)	median, 111	median, 39	median, 110
ALT (1000 unit/L)	median, 100	median, 36	median, 110
Creatinine (mg/dl)	median, 1.2	median, 1.3	median, 1.1
Urea (mg/dl)	median, 34	median, 34	median, 31
Received platelet transfusion	2 (6.9%)	0 (0%)	2 (10%)

DF: dengue fever; W/SD: warning signs or severe dengue; ALT: alanine aminotransferase; AST: aspartate aminotransferase.

The most common clinical features observed in this series were fever (26 [89.7%] patients), myalgia (19 [65.5%] patients), and drowsiness (15 [51.7%] patients), followed by headache (14 [48.3%] patients) and hemorrhagic manifestations such as petechiae and epistaxis (14 [48.3%] patients), nausea or vomiting (10 [34.5%] patients), and abdominal pain (9 [31.0%] patients). All 29 patients (1.43% of 2020 patients with confirmed dengue) underwent chest CT due to respiratory symptoms, most frequently dyspnea (11 [37.9%] patients).

Thrombocytopenia occurred in 23/29 (79.3%) patients. Total leukocyte counts were decreased in 17/29 (58.6%) patients. AST and ALT levels were elevated in 15/18 (83.3%) and 13/18 (72.2%) patients, respectively. Creatinine and urea levels were elevated in 11/27 (40.7%) and 24/27 (88.9%) patients, respectively.

### CT Findings

Chest CT findings were normal in 12/29 (41.38%) patients and abnormal in 17/29 (58.62%) patients (5 with DF, 12 with W/SD). Eleven of 17 patients with abnormal chest CT findings (3 with DF, 8 with W/SD) had parenchymal abnormalities. PE was the sole finding in 6/17 patients.

**Figure 2:**
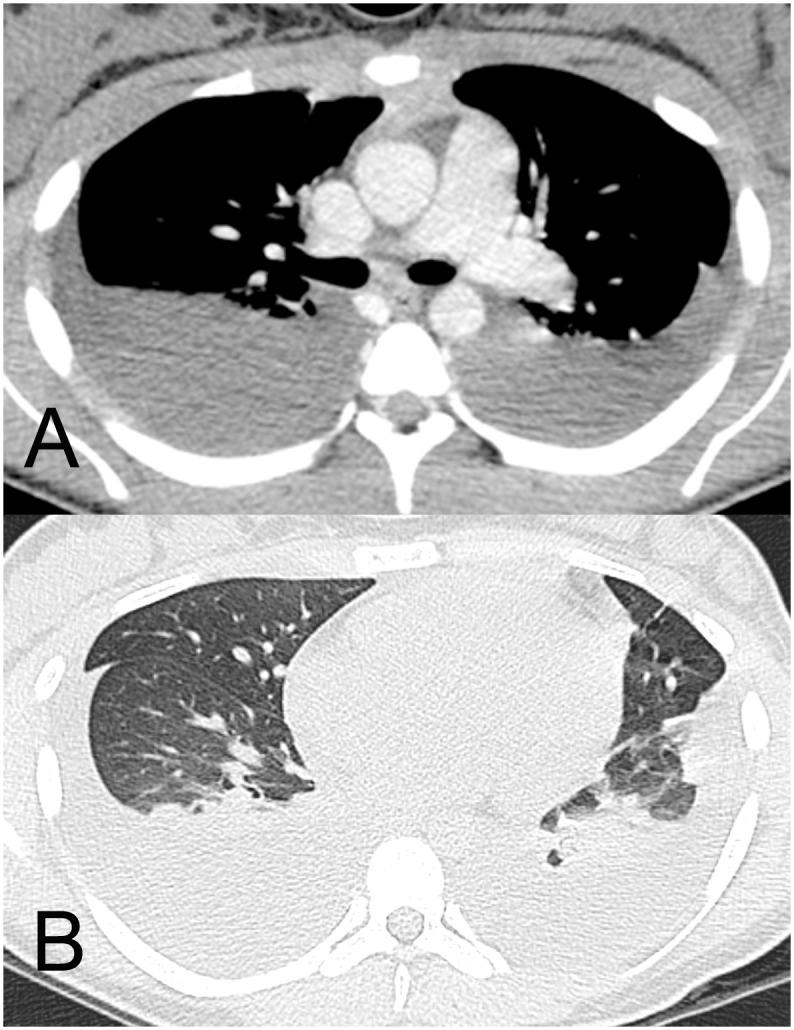
A 16-year-old girl with a confirmed diagnosis of dengue and reduced exercise tolerance and breathlessness. CT images show large bilateral pleural effusion (a, mediastinal window) and no abnormality of the lung parenchyma other than compressive atelectasis (b, lung window).

PE was detected in 16 (55.17%) patients (5 with DF, 11 with W/SD). It was bilateral in 13 cases and unilateral (right-sided) in 3 cases. PE was small in eight patients (four with DF, four with W/SD), moderate in four (one with DF, three with W/SD), and large in four patients (all with W/SD; Fig. 2). No patient with DF had large PE.

**Figure 3:**
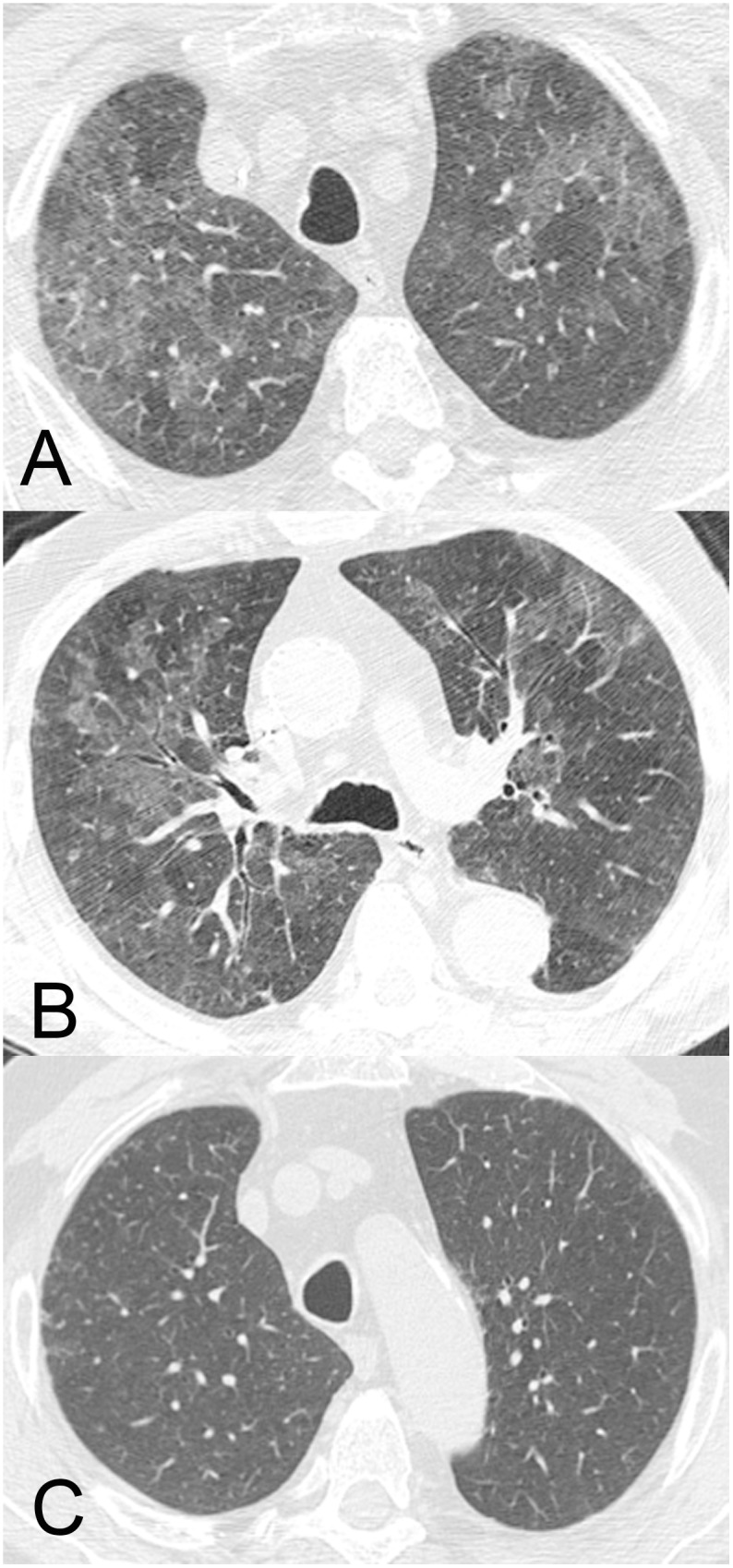
A 78-year-old man with a confirmed diagnosis of dengue. CT images (a, b) show moderate and diffuse ground-glass opacities with no specific distribution in the lungs.

**Figure 4:**
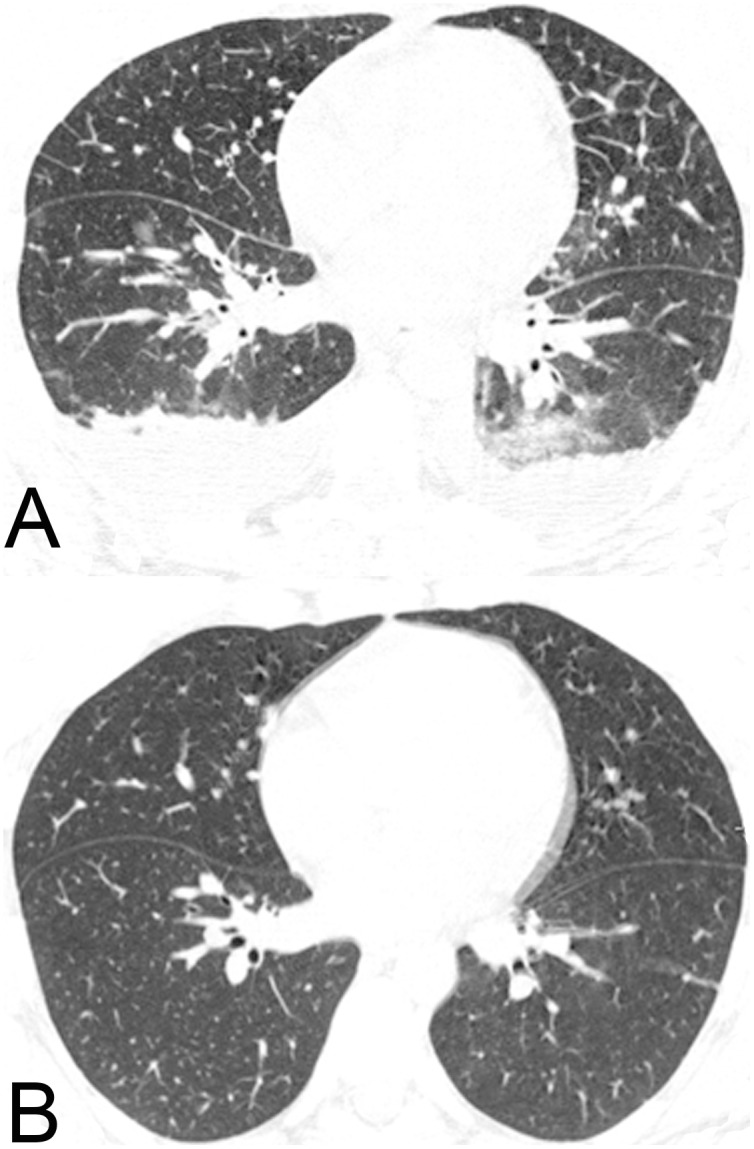
A 30-year-old woman with a confirmed diagnosis of dengue and respiratory failure requiring non-invasive ventilation. CT image acquired 6 days after the onset of clinical symptoms (a) shows interlobular septal thickening, mild ill-defined ground-glass opacities, and peribronchovascular interstitial thickening. Bilateral pleural effusion is also present. Complete resolution of the parenchymal abnormalities was observed on a CT images (b) obtained 7 days later.

Lung parenchymal involvement was observed in 11 patients; it was bilateral in 9 (2 with DF, 7 with W/SD). The most common finding was GGO (eight patients [three with DF, five with W/SD]; Fig. 3), followed by consolidation (six patients [two with DF, four with W/SD]). Airspace nodules and interlobular septal thickening were observed in two patients each (two with W/SD and one with DF, one with W/SD, respectively) and peribronchovascular interstitial thickening occurred in one patient with W/SD (Fig. 4).

GGO and consolidation had no specific distribution. Smooth interlobular septal thickening was mild and located predominantly in the upper lobes in one patient with DF; in a patient with W/SD, it was moderate and distributed predominantly in the lower zone. Peribronchovascular interstitial thickening, noted in only one patient (with W/SD), was intermediate in severity and occurred in the middle and lower zones. Mild airspace nodules were observed in 2/20 patients with W/SD and occurred in the middle zone. No patient with DF presented airspace nodules. No specific axial distribution was observed.

Among all patients with lung involvement, the extent of disease was considered mild in four cases (two with DF, two with W/SD), moderate in six cases (one with DF, five with W/SD), and severe in only one case (with W/SD). The extent of disease tended to be greater in patients with W/SD than in those with DF, but this difference was not significant.

### Histopathological Analysis of Lung Tissue Obtained from Fatal Cases

**Figure 5:**
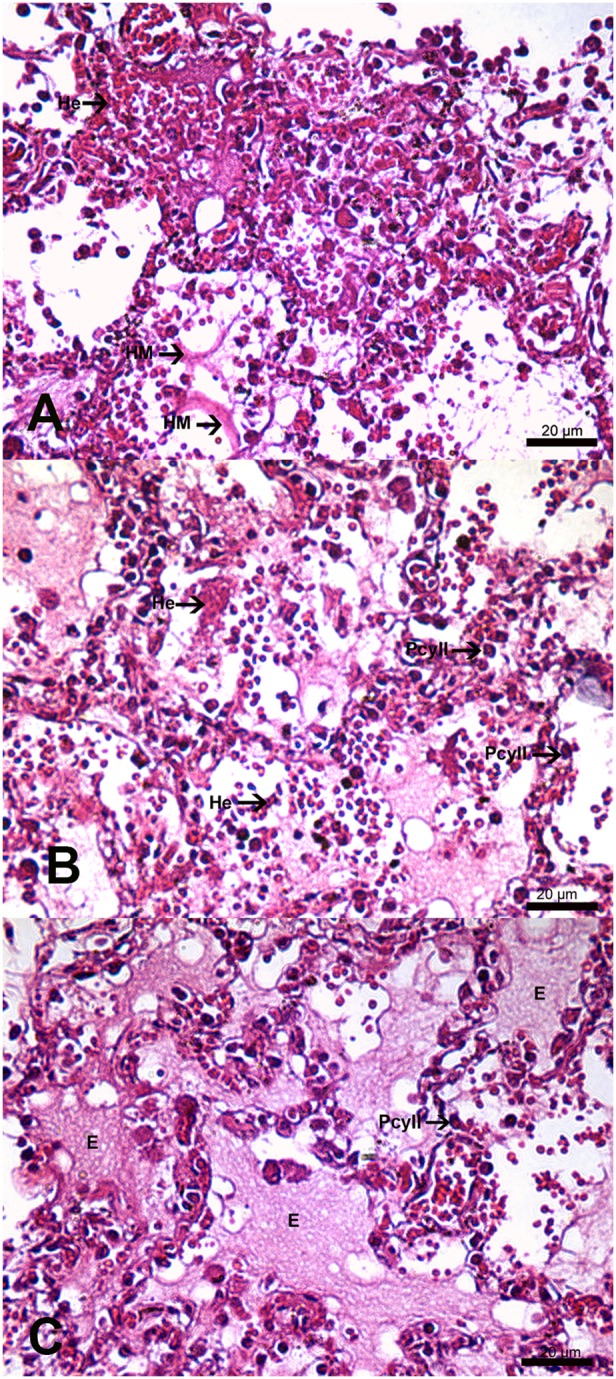
Histopathological analysis of lung sections from fatal dengue cases (a, b, c), stained with hematoxylin and eosin, showed pulmonary alterations, including alveolar septal thickening, hyaline membrane formation (HM), hemorrhage (He), edema (E), and the presence of type II pneumocytes (PcyII).

The human lung tissues analyzed in this study were obtained from four fatal dengue cases. All patients, ranging in age from 21 to 63 years, presented fever, myalgia, and hemorrhagic manifestations. Dengue diagnoses were confirmed by serum IgM antibody positivity. Necropsies revealed microscopic findings related to lung damage, such as pulmonary congestion, alveolar edema, and hemorrhage, in all cases. Histopathological analysis of the lung tissues revealed alveolar septal thickening, focal hyaline membranes, hyperplasia of type II pneumocytes, and circulatory injuries, including diffuse areas of hemorrhage and edema (Fig. 5).

## Discussion

In this large series of adults with serologically proven dengue infection, we observed that severe involvement of the respiratory system was uncommon and that the most frequent chest CT findings, such as PE and signs of pulmonary edema, were probably associated with increased vascular permeability. Post-mortem histopathological analysis showed interstitial edema, diffuse alveolar congestion, and hemorrhage in the lungs.

In this cohort of 2020 dengue cases confirmed at three tertiary hospitals in Rio de Janeiro, Brazil, 1.43% of patients underwent chest CT due to respiratory manifestations. This frequency is similar to that found by Halsey et al. [14] in a series of patients from South America (1.3% of patients had dyspnea). Several authors [14], [15] have reported variable frequencies of respiratory symptoms in patients with dengue, but these symptoms are generally mild and mainly affect the upper airway. Mustafa et al. [15] reported that patients with DF were less likely to have respiratory symptoms than were patients with non-dengue febrile illnesses. Severe involvement of the lower respiratory system is uncommon in dengue.

In our series, 58.6% of patients who underwent chest CT showed abnormalities; PE was the major finding, observed in 55% of patients with abnormalities and constituting the sole finding in 35% of these patients. Wang et al. [16] reported a similar percentage (54.7%) of abnormal chest radiographic findings in patients with dengue. Variable frequencies of PE, ranging from 16% [17] to 100% [18] of patients, have been reported in studies in which chest X-rays and sonography were used to evaluate dengue infection. However, few studies have been performed in adults and most have used chest X-rays [16], [17]. Higher incidences of PE have been demonstrated in studies conducted in children and those using sonography [19], [20]. Some authors have shown higher frequencies of PE in patients with SD [19], [20]; for example, Balasubramanian et al. [19] found that PE was twice as frequent in children with SD than in those with DF. In our series, the frequency of PE was 55% in both groups, but moderate or large PE was present more often in patients with W/SD. Some authors have correlated larger PE with disease severity [20], fluid management [6], and viral serotype [21]. All patients in our study had right-sided or bilateral PE and no patient had left-sided PE, as observed by other authors [16], [18].

To our knowledge, only one original study has evaluated lung involvement in adult patients with dengue [16]. In that study, the authors retrospectively reviewed 468 chest X-rays to determine the presence of infiltration and PE. No chest X-ray showed both infiltration and PE, and infiltration was the sole finding in 42.6% of cases. In our study, 65% of patients with abnormal chest CT findings had lung involvement. This difference is probably related to the higher sensitivity of CT compared with plain radiography for the evaluation of lung parenchymal abnormalities. Our data also showed a tendency for mild or moderate lung involvement in dengue, which could explain the lower incidence of pulmonary infiltrates found on X-rays [16]. Severe pulmonary involvement, however, has been described in a few case reports [9], [10], [22].

Pulmonary involvement by dengue virus has been demonstrated to be an uncommon finding in fatal cases [8]. In such cases, viral antigens have been detected in different human cells, including alveolar macrophages and endothelial cells in the lungs [23]. Histopathological analysis of specimens from four fatal cases in our study revealed thickening of the alveolar septa and the presence of large areas of hemorrhage and interstitial edema, as well as hyaline membrane formation and hyperplasia of type II pneumocytes. Extensive lung involvement in fatal cases, especially in association with lung hemorrhage, is a terminal event in dengue and is probably associated with uncontrolled infection and shock [24]. Histopathological findings in our cases were extensive, whereas CT images showed mainly mild/moderate disease extent, probably reflecting differences in severity.

Breathlessness may occur in patients with DF due to PE, acute respiratory distress syndrome, pulmonary hemorrhage, pneumonia, and shock [25]. PE is the most frequent cause of breathlessness in patients with dengue [26]. In our series, lung parenchymal involvement was characterized mainly by mild or moderate GGO and consolidation and, infrequently, by airspace nodules, interlobular septal thickening, and peribronchovascular interstitial thickening, probably reflecting edema. Recent publications discussed the computed tomography findings of 3 patients with pulmonary hemorrhage syndrome associated with dengue fever. All patients presented similar aspects, with extensive areas of consolidation and/or ground glass opacities diffuse in both lungs [10], [22], [27].

Fluid accumulation in the lungs may be the result of changes in pulmonary venous hydrostatic pressure and/or increased permeability of capillary endothelium. The CT features of these two processes overlap considerably and are often difficult to distinguish [28]. In a febrile illness, the presence of plasma leakage, such as PE or ascites, suggests the diagnosis of dengue [26]; nonetheless, the presence of extensive lung abnormalities should lead physicians to consider other diagnostic possibilities, even during dengue outbreaks [29].

Clinical and radiological distinction between dengue and other infections associated with diffuse pulmonary hemorrhage may also be challenging. In immunocompetent patients, the most important infectious diseases for the differential diagnoses include influenza A (H1N1), dengue, leptospirosis, and malaria [27], [30]–[32].

Several laboratory diagnostic tests may be used to detect the presence of influenza viruses in respiratory specimens. Real-time reverse-transcriptase polymerase chain reaction (rRT-PCR) has the highest sensitivity and specificity [27]. Also, a pattern of extensive or diffuse ground-glass opacities and consolidations with a primarily peribronchovascular or subpleural distribution may be strongly related to influenza A (H1N1) infection [27], [32]. The diagnosis of leptospirosis is based on clinical findings, a history of direct or indirect exposure of mucous membranes or abraded skin to infected animals or their urine, in endemic areas, especially in the context of urban floods or other immersion in contaminated water, and positive serologic tests [27], [30], [31]. In patients living or having traveled in endemic areas, malaria should be considered as a possible cause of respiratory failure of obscure etiology. Parasites may be detected on thick and thin peripheral blood smears. Thick smear examination facilitates the quantification of parasitemia [27].

This study has some limitations. As a retrospective study, the CT indications were based on clinical manifestations and not on previously established criteria, which could have led to the underestimation of the prevalence of respiratory system involvement in this series. Despite the limitations inherent to retrospective studies, our series included a large number of patients with serologically confirmed disease and is the first original study to use CT for the assessment of pulmonary involvement in such patients.

In conclusion, dengue has become very prevalent in tropical and subtropical regions, affecting hundreds of thousands people in large urban areas. Lung abnormalities are infrequent in dengue and CT findings probably reflect increased vascular permeability. Extensive pulmonary involvement is related to severe forms of the disease and should lead physicians to consider other diagnostic possibilities, even during dengue outbreaks.
